# Perspectives on Medical School Admission for Black Students Among Premedical Advisers at Historically Black Colleges and Universities

**DOI:** 10.1001/jamanetworkopen.2024.40887

**Published:** 2024-10-23

**Authors:** Jasmine Weiss, Max Jordan Nguemeni Tiako, Ngozi D. Akingbesote, Danya Keene, Lilanthi Balasuriya, Mona Sharifi, Inginia Genao, Darin Latimore

**Affiliations:** 1 Division of General Pediatrics and Adolescent Medicine, Department of Pediatrics, University of North Carolina School of Medicine, Chapel Hill; 2 Department of Medicine, Brigham and Women’s Hospital, Boston, Massachusetts; 3 Harvard Medical School, Boston, Massachusetts; 4 Urban Health Lab, Perelman School of Medicine at the University of Pennsylvania, Philadelphia; 5 Department of Cellular and Molecular Physiology, Yale School of Medicine, New Haven, Connecticut; 6 Section of Endocrinology and Metabolism, Department of Internal Medicine, Yale School of Medicine, New Haven, Connecticut; 7 Department of Social and Behavioral Sciences, Yale School of Public Health, New Haven, Connecticut; 8 National Clinician Scholars Program, Yale School of Medicine, New Haven, Connecticut; 9 Department of Pediatrics, Yale School of Medicine, New Haven, Connecticut; 10 Department of Medicine, Penn State College of Medicine, Hershey, Pennsylvania; 11 Department of Internal Medicine, Yale School of Medicine, New Haven, Connecticut

## Abstract

**Importance:**

There remains a lack of representation of Black physicians in the physician workforce despite decades of national efforts to increase diversity. Historically Black Colleges and Universities (HBCUs) contribute to increasing representation in the workforce in the US. There is limited literature exploring the experiences of HBCU premedical students navigating the journey to medical school.

**Objective:**

To examine barriers to and facilitators of medical school matriculation among Black students by exploring the perspectives of premedical student advisers at HBCUs.

**Design, Setting, and Participants:**

In this qualitative study, HBCU advisers participated in a focus group in October 2019 or in semistructured interviews from January 2020 to March 2021. Using purposive sampling, premedical student advisers were recruited from different educational backgrounds and geographic locations and conducted 1-on-1 interviews. Data analysis was performed from March 2021 to March 2022.

**Main Outcomes and Measures:**

The main outcome was the experiences of HBCU premedical students navigating to medical school from the perspective of premedical advisers. An inductive approach to generate codes from the interviews and the focus groups was applied by reviewing transcripts and noting concepts and themes associated with advising strategies and student experiences.

**Results:**

Among the 26 participants, 18 (69.2%) were female, 13 (50.0%) had a doctorate degree, and 8 (30.8%) had more than 10 years of experience as advisers. Participants described 3 major themes: (1) the complex institutional relationship dynamics between undergraduate HBCUs and medical schools, (2) concerns about preferential treatment for students from predominantly white undergraduate schools when accessing clinical shadowing opportunities, and (3) the “it takes a village mindset” emphasizing family and peer involvement.

**Conclusions and Relevance:**

In this qualitative study, barriers to medical school matriculation included concerns for the preferential treatment of students from predominantly white institutions when engaging with medical schools and a lack of access to clinical opportunities for HBCU students. The findings suggest that medical schools should continue to build collaborative partnerships with HBCUs to ensure equitable access to opportunities that prepare students for medical school.

## Key Points

**Question:**

How do premedical student advisers at Historically Black Colleges and Universities (HBCUs) describe the barriers to and facilitators of Black premedical students successfully navigating to medical school?

**Findings:**

In this qualitative study of 26 HBCU premedical student advisers, barriers to and facilitators of students successfully matriculating to medical school were complex relationships between undergraduate HBCUs and medical schools, concern for preferential access to clinical opportunities for students of predominantly white undergraduate schools, and the importance of support networks.

**Meaning:**

The findings suggest that an opportunity exists for medical schools to build stronger relationships with HBCU undergraduate institutions, which has implications for increasing representation among medical school applicants and the physician workforce.

## Teaser

This qualitative study assesses perspectives of premedical student advisers from Historically Black Colleges and Universities (HBCUs) on the barriers to and facilitators of medical school matriculation among Black students.

## Introduction

In the US, health disparities persistently affect the Black community, leading to worse outcomes for many major health indicators, including infant and maternal mortality.^1,2^ Recent evidence highlights that Black physicians can play a role in mitigating these disparities and other survival-related outcomes.^3,4^ The underrepresentation of Black physicians in the workforce may exacerbate health disparities by impeding access to care given that individuals from historically underrepresented backgrounds are more likely to practice in underserved communities^5^ and care for publicly insured and uninsured patients.^6,7^ Despite their effects on access to care in marginalized communities and various health outcomes, Black physicians remain severely underrepresented in the physician workforce.^8^ The Association of American Medical Colleges (AAMC) defines individuals as underrepresented in medicine (URiM) if they belong to racial and ethnic groups with less representation in the medical profession relative to the general population.^9^ According to 2023 US Census Bureau statistics, there were 45.3 million Black or African American individuals in the US, which represented 13.6% of the US population.^10^ Yet, only 5.7% of physicians in the US self-identified as Black.^8^

Given the recent US Supreme Court decision on race-neutral admissions and the revised Liaison Committee on Medical Education (LCME) standard (Element 3.3) allowing for more leniency in reporting diversity outcomes, there is concern that these changes may further contribute to a decline in racial and ethnic diversity within the physician workforce.^11,12^ Previous studies have demonstrated an association of state anti–affirmative action policies with decreases in medical student enrollment of URiM students at public medical schools, showing a 32% decline in URiM medical school enrollment following the passage of such policies.^13^ This recent federal decision raises significant concerns about the potential for similar nationwide declines in URiM student enrollment.

Historically Black Colleges and Universities (HBCUs) play a crucial role in increasing the diversity of the physician workforce and in training Black physicians.^14,15,16^ Undergraduate HBCUs account for 25% of all Black graduates receiving science, technology, engineering, and mathematics degrees and 25% of all Black graduates receiving baccalaureate degrees despite making up only 3% of all colleges and universities in the US.^17^

HBCUs also make a significant contribution to the workforce of Black physicians.^14,18,19^ According to the AAMC, 9.8% of Black medical school graduates in 2019 completed their undergraduate training at HBCUs.^18^ HBCUs are not only a pathway of incoming medical school students, but they also overproduce leaders in academic medicine. HBCU medical schools comprise only 2.4% of all medical schools, but HBCU medical school graduates constitute 31% of all Black department chairs in academic medicine, 10% of all Black academic faculty, and 10% of all Black medical school students.^16^

Despite the known benefits of HBCUs, there is a paucity of literature exploring how Black HBCU undergraduate students navigate the process and successfully gain admission to medical school and how premedical advisers support them. In this qualitative study, we aimed to identify barriers to and facilitators of medical school matriculation among Black students by exploring the perspectives of premedical advisers at HBCUs. Greater insights into the barriers to and facilitators of medical school matriculation for Black HBCU students may help identify effective strategies to increase the numbers of Black individuals entering the physician education training pathway, with the goal of increasing the number of practicing Black physicians from 5.7% to parity with the Black US population at 13.6%.^8,10^

## Methods

### Participants, Sampling, and Recruitment

In this qualitative study using purposive sampling, we recruited advisers whose role allowed for frequent interactions with students seeking admission to medical school.^20^ During the Yale School of Medicine’s First Look Immersion Program on October 10 and 11, 2019, the study team conducted 1 in-person semistructured focus group. This event, sponsored by the Office of Diversity, Inclusion, Community Engagement, and Equity, attracted premedical advisers and students from HBCUs to share more knowledge about the Yale School of Medicine as an institution and the medical school admissions process in general. After the event, the study team lead investigator (J.W.) conducted additional 1-on-1 interviews with HBCU premedical advisers from other institutions using video conferencing from January 2020 to March 2021. Using information from HBCU websites, the study team identified additional premedical advisers to participate in the study. Two participants from the focus group also agreed to participate in 1-on-1 interviews. We obtained verbal consent from all study participants, which included permission to publish anonymous quotations. Two advisers who were contacted for interviews declined study participation. The Yale institutional review board deemed this study non–human participant research. This study reports our findings using the Consolidated Criteria for Reporting Qualitative Research (COREQ) reporting guideline.^21^

### Data Collection

We developed a semistructured interview guide to evaluate current practices for student advising and how advisers perceived and approached obstacles that students may face along the pathway to medical school (Box). Two physician researchers assisted with interview guide pilot testing. After the initial focus group, the guide was revised based on participant responses and used by the lead investigator (J.W.) to conduct 45- to 60-minute semistructured interviews. All interviews were audio recorded and transcribed verbatim. Each study participant also completed a survey to collect their demographic data (including number of years wanting to serve as a premedical adviser and advisers’ level of education). Self-reported race and ethnicity categories of study participants included Asian, Black or African American, Hispanic or Latino, and White. Interviews were completed after achieving thematic saturation (ie, when no new concepts emerged from data collection and all interview data were assigned to codes and categorized into preexisting categories).^22^

Box. **Interview Guide**

1. To begin, can you each briefly describe how you became a premed adviser? Reminder to state your study ID number prior to your responses. (Icebreaker.)
2. Describe an example of a student that wanted to attend medical school but didn’t go. Please elaborate on your example.
3. Describe some of the institutional resources that you used to assist your student during this time.
4. Are there any specific obstacles that discourage a sizeable proportion of students at your college from pursuing medical school? Please elaborate on your example.
5. Describe an example of a typical student that is successful at gaining acceptance to medical school.
6. Describe some of the institutional resources that you used to assist your student during this time.
7. How, if at all, do you think attending an HBCU impacts a student pursuing medical school? Describe a specific example.
8. Based on your experiences, how do students choose what schools to apply to? Can you share a specific story?
9. During your time as an adviser, has your institution had any specific relationships with medical schools?
  - How did those relationships form?
  - Have they changed over time?
10. Which medical schools come to your school to recruit students?
  - Local?
  - State?
  - Research intensive?
  - Ivy League?
11. What is the most challenging part of your job? Or what is one thing you love about your job?

Abbreviations: HBCU, Historically Black College and University; ID, identification; premed, premedical.

### Data Analysis

Data analysis was conducted from March 2021 to March 2022. The study team used an inductive approach to generate codes from the interviews and the focus groups by reviewing transcripts and noting concepts and themes related to advising strategies and student experiences.^23^ Both J.W. and M.J.N.T. are graduates of undergraduate HBCUs and predominantly white institution (PWI) medical schools. They are also faculty members with expertise in workforce diversity, equity, and inclusion; advocacy; and medical education. Other team members included physicians, researchers, faculty members, and medical school chief diversity officers. All team members had expertise in qualitative research, health disparities, medical education, and diversity, equity, and inclusion. Some members of the study team (J.W., L.B.) coded and discussed the focus group together to ensure interrater reliability and coded the next 5 interviews independently line by line, meeting regularly to discuss any new codes or redundant codes, and then generated a finalized codebook. Other team members (J.W., N.D.A.) then applied the finalized codebook to all remaining interviews, meeting frequently throughout the process. We analyzed the data using a thematic analysis to generate themes. We used NVivo software, version 12 (QSR International) for data storage and organization.

## Results

### Participant Characteristics

Twenty-six premedical advisers from HBCUs participated in this study (18 female [69.2%] and 8 male [30.8%]). Table 1 highlights the study participants’ demographic characteristics, institutional region, educational background, and years of experience as advisers. Some premedical advisers had other roles at the university, including as course instructors. Among the 26 participants, 4 (15.4%) self-identified as Asian, 19 (73.1%) as Black or African American, 1 (3.8%) as Hispanic or Latino, and 2 (7.7%) as White. Thirteen participants (50.0%) had a doctorate degree, and 8 (30.8%) had more than 10 years of experience as a premedical adviser.

**Table 1.** Demographics of Participating Premedical Advisers at Historically Black Colleges and Universities

Characteristic	Participants, No. (%)
Sex	
Female	18 (69.2)
Male	8 (30.8)
Age range, y	
25-34	1 (3.8)
35-44	8 (30.8)
45-54	6 (23.1)
≥55	11 (42.3)
Race and ethnicity	
Asian	4 (15.4)
Black or African American	19 (73.1)
Hispanic or Latino	1 (3.8)
White	2 (7.7)
Educational level	
Master’s degree	9 (34.6)
Professional degree	4 (15.4)
Doctorate degree	13 (50.0)
Formal training	
Yes	4 (15.4)
No	22 (84.6)
Time wanting to serve as premedical adviser, y	
<1	1 (3.8)
1-2	3 (11.5)
3-5	9 (34.6)
6-10	5 (19.2)
>10	8 (30.8)
Institutional region	
Southern division	
East South Central division	8 (30.8)
South Atlantic division	14 (53.8)
West South Central division	2 (7.7)
Northeast or Mid-Atlantic division	2 (7.7)

### Themes

Participants described 3 major themes concerning barriers to and facilitators of premedical students successfully gaining acceptance to medical school: (1) complex institutional relationship dynamics between undergraduate HBCUs and PWI medical schools, (2) concern for institutional bias in access to clinical opportunities, and (3) an “it takes a village mindset” (ie, family and peer involvement to incentivize student accountability). Table 2 provides illustrative quotations from each theme.

**Table 2.** Themes With Illustrative Quotations From Premedical Advisers

Theme	Illustrative quotation
Complex institutional relationship dynamics between undergraduate HBCUs and medical schools: trust, mistrust, and collaboration	They’re quick to count people out…but it’s so many people who don’t even get a chance because…they’re looking at [the] MCAT. We talk about holistic. It’s holistic after the MCAT.
	Because of the population that we work with, when we are speaking with individuals who try and discredit the students and what their potential future could be just by a snapshot, it makes it very difficult because we have all the stories of success.
Preferential treatment of PWI students for clinical shadowing opportunities	This [less access to shadowing opportunities than nearby PWI students] is something that needs to be looked at…some action needs to be taken. Because this is one of the areas that is really hurting our students because our students are not getting those same types of experiences.
	So we’re having [students] try and take advantage of that [local hospital CNA training program]. But that is allowing them employment as a way of overcoming the need to get shadowing from a volunteer clinician.
“It takes a village mindset”: family and peer involvement to incentivize student accountability	We ask parents to contribute financially. We want…[them] to see what we are providing for the students…. If you want to…help us out, we have a fund that we call the [fund name], and a lot of parents contribute during that time or during the white coat ceremony. So they will make a contribution to the academy to show their buy-in and their appreciation for what we do.
	We asked a couple of our students that just got accepted to come back and talk about [the] MCAT…. One of the students [facilitated] a workshop and talked about tips, technique, [and] test-taking strategies…because there’s a lot more to this test than just a normal test.
	Attending [an] HBCU is beneficial because [it] is a nurturing environment. In my dissertation, I examined factors that contributed to the success of African American biology majors at HBCUs, and the 2 factors that stood out [were] experiences with faculty and the campus environment to their success.

Abbreviations: CNA, certified nursing assistant; HBCUs, Historically Black Colleges and Universities; MCAT, Medical College Admission Test; PWI, predominantly white institution.

#### Complex Institutional Relationship Dynamics Between Undergraduate HBCUs and Medical Schools—Trust, Mistrust, and Collaboration

Advisers reported substantial variability in the degree to which PWI medical schools engaged with undergraduate students. Some relationships were viewed as exploitative.

We’re very well aware that the AAMC has told them that they need to have diversity admissions at their institution and how they will come to HBCUs to pimp us on pipe dreams that really aren’t going to allow us to see the traction necessary to increase the number of students…and so they come, they get the students excited, but there’s no real intention of accepting them unless they have 90th percentile scores. And so it looks great for them–yep–but it still does us no service.

Many advisers highlighted that medical schools would come under the guise of seeking all students who would be a good fit for their program. However, they ultimately would only seriously consider the most competitive applicant, often based on test scores, as opposed to taking a holistic view of an applicant.

Come to our school. We want Black people. Right. So they say it a lot…But you PWI want the ones that everybody wants. That’s right. The one with that MCAT [Medical College Admission Test] that’s way up there, 90th percentile, you want that person. Everybody’s vying for that one person. No, we’ve got to think about this differently if we’re really committed to increasing numbers of underrepresented minorities, especially Black students.

Some premedical advisers reported tempering their expectations of medical schools that come to recruit students, indicating that they had more faith in medical schools with a track record of admitting their students in the past.

I’ve started…to pull down the metrics on how many of our students have applied [to your PWI medical school] and how many they’ve already accepted. So if you haven’t shown any promise of doing that previously, I don’t have any confidence that you’re going to do it afterward.

In contrast, some advisers perceived that their medical school relationships were more genuine and of mutual benefit. Particularly, a few advisers reported strong relationships with osteopathic schools, Caribbean schools, and HBCU medical schools. As 2 advisers noted:

Our state schools have been pretty supportive of accepting our students through the pipeline…. They offered us a guaranteed interview for all of our students. We have at least 3 students, it seems, that gets accepted there each year, and it’s an osteopathic medical school. We’ve had really good relationships with those medical schools.

One of the Caribbean schools that’s been really good about accepting our students…. The students that have attended have come back and told us it was a positive experience. And so if they don’t get into one of the schools here, we say, “Let’s look at [school name].”

#### Preferential Treatment of PWI Students for Clinical Shadowing Opportunities

Some premedical advisers reported concerns that local medical facilities gave priority treatment to PWI undergraduate students for access to clinical experiences, which were needed for students to be as competitive as possible when applying to medical school.

I’m 5 minutes away from another PWI…and they have agreements with everyone. We have 2 hospitals and…a number of health care facilities, assisted living facilities…they have agreements with all of them…and many of their students, because they have these agreements, their students are already in place. They already have a network, pipeline system set up for their students.... So for many of the students at my particular institution, it’s impossible for them to be able to secure any type of shadowing experience.

Some advisers attributed the lack of access to opportunities for HBCU students to the limited supply of physicians who identified as historically URiM. Respondents anecdotally described more of a willingness of URiM physicians to create shadowing opportunities for URiM students. “With very few African American doctors, we don’t have the access that some other groups have to get into the hospital to get that clinical experience.”

Some HBCU advisers viewed student placement for clinical experiences competitively when considering students’ opportunities and the opportunities for students at PWIs.

There’s a PWI 20 minutes from my institution. And this is a major obstacle for me. Trying to get my premed students in the same organization that the students go to for clinical rotations and for clinical hours. It has been a constant struggle for me to get my students there…. I’m almost in stalker mode…. Literally stalker mode. Trying to get this to happen for my students, so. And then you don’t want to overload the ones that are providing access.

While access to these direct opportunities posed a challenge for some participants, others created alternative strategies to ensure that students could use clinical experiences to receive credits that would contribute to their progress toward graduation.

When we saw some of the same challenges, [we] developed a major focused in health and health sciences and incorporated an internship course in the curriculum…. By institutionalizing it, it has helped us quite a bit.

Others encouraged students to get other health sciences certifications to serve as both employment opportunities and clinical experiences.

Dual enrollment—many of the students are graduating with their certified nursing assistant, phlebotomy, and other certifications that allow them the ability to work in those capacities versus actually having a [fast food] job and then trying to shadow on the side.

Table 3 includes additional recommendations from the study participants to combat the barriers that students face.

**Table 3.** Key Recommendations From Premedical Advisers

Recommendation	Description	Illustrative quotation
Create a national physician shadowing database	Develop a centralized platform to connect premedical students with physicians for shadowing opportunities across different geographic locations, disciplines, and subspecialties.	I would like to encourage a national list so we can identify some people [providing shadowing] from state to state.... Because getting shadowing experiences for students [has] been a challenge nationwide.
Strengthen institutional partnerships between PWIs and HBCUs	Foster institutional collaborations between HBCUs and PWIs to increase access to mentorship, research, clinical opportunities, and other resources for HBCU students. Consider joint networking events, research symposiums, tutoring opportunities, or simulation exercises.	I have a good affiliation with the PWI close by and they’re very excited about our [revitalized MAPS program]. They are excited because they realize how they have so few minorities. If you want these students, you go where the Black students are, and that is at [HBCUs].
Increase summer program opportunities	Provide HBCU students with medical school exposure through summer programs, both in state and out of state.	Being able to have opportunities with schools through summer programming...for students to be able to experience medical school, especially outside of their comfort zone…. Because we tell students, “Cast a wide net when you’re applying to medical school. But before that, cast a wide net with summer programs.”
Create opportunities for peer and near-peer mentorship and support	Create opportunities for students to connect across PWIs and HBCUs to premedical students, medical students, residents, and fellows. Foster relationships between PWI medical students and nearby HBCU undergraduate students.	I’ve had some of my students, my alumni who [have gone] to [or] finished med school…come back and speak this year.... That’s the best part…[the students] have a chance to talk to them and hear what route they took to get where they were.

Abbreviations: HBCUs, Historically Black Colleges and Universities; MAPS, Minority Association of Premedical Students; PWIs, predominantly white institutions.

#### “It Takes a Village Mindset”—Family and Peer Involvement to Incentivize Student Accountability

Many advisers recognized the importance of family, peers, and alumni in a student’s navigation to medical school. Some found creative opportunities to include families and peers into the academic process for students. One participant noted:

At our institution, we set up sort of a checklist of activities in which students need to complete at every level, and it’s really telling when the parents come, and we ask students, “Well, let’s pull your folder to see where you are,” and of course, when the parents see they only have 3 or 4 checks on this list of 20 things, then it starts the conversation.

Advisers expressed the importance of on-campus opportunities for families to engage and familiarize themselves with the physician career path. One participant stated:

[We created the] first-ever white coat prehealth induction ceremony…. That was a signal to the support systems, to grandma, to auntie, to everyone. “You know what, this is the career path I want to take.” ….We see the white coats on campus, walking around, moving around. And now, grandma is calling saying, “Hey. How’s my baby doing up there?” Okay, so it’s sort of a holistic approach to advising.

Participants acknowledged the need for multiple sources of encouragement and accountability in supporting the students in contrast to the adviser serving as the singular source of accountability. One participant remarked:

At the beginning of each year, the new cohorts, we have parent orientation.... It’s mandatory that they attend orientation. And that’s where the director will lay out his expectations for the students, and what he expects from the parent.

By providing families with the awareness of the steps needed for successful medical school preparation, families became another source of accountability for students.

## Discussion

In this qualitative study exploring the perspectives of premedical advisers at HBCUs nationally, we found that complex institutional relationship dynamics between undergraduate HBCUs and medical schools coupled with concerns for the preferential treatment of PWI students in offering shadowing experiences were perceived as important barriers to medical school entry among HBCU students. Among strategies to mitigate these biases and to facilitate progress toward successful enrollment in medical school, advisers highlighted the importance of innovative approaches to cultivate clinical opportunities and to engage students’ familial and social networks to help them successfully navigate to medical school.

HBCUs have been instrumental to the training of Black physicians in the US. With the historic 2009 requirements of the medical school LCME diversity standard, it would be intuitive to expect PWI medical schools in close geographic proximity to HBCUs to strengthen their relationships with HBCUs as part of their diversity efforts.^24^ Although some partnerships exist between undergraduate HBCUs and neighboring PWI medical schools for summer enrichment or shadowing opportunities, our findings suggest that these organizational partnerships are perceived by many HBCU premedical advisers to infrequently result in increased medical school admissions, even for well-qualified, competitive HBCU applicants.

Beyond the lack of reciprocity due to perceived institutional status, it is possible that PWI medical schools discriminate against Black HBCU students, as they may be perceived as less racially acceptable because attending an HBCU signals racial salience,^25^ defined as “the degree to which race and Black culture are [perceived to be] important to an individual.”^26^ Sociological research has shown that racial salience shapes how racial and ethnic minority groups are hired, promoted, and accepted within organizations.^25,26^ At the college admissions level, for example, research has shown that White admissions counselors are more responsive to Black students who appear less concerned about race and racism.^27^ In the medical school pathway context, admissions officials seeking to increase their schools’ enrollment of Black students may similarly favor Black students from PWIs. Furthermore, there is a concern that with the revised LCME Element 3.3 standard, PWIs will place less emphasis on creating new partnerships with HBCUs given that schools have more leniency with measuring their recruitment outcomes for racial and ethnic diversity.^12^

Another institutional factor that may contribute to the perception of preferential treatment is that most medical schools are part of a larger university ecosystem and are affiliated with PWIs. In geographic locations where there is an undergraduate HBCU and a PWI medical school, the undergraduates enrolled at the PWI may have an inherent advantage over their HBCU counterparts through institutional affiliation and physical proximity. This proximity may impact the HBCU students’ access to enrichment opportunities that strengthen their medical school application, such as shadowing opportunities, research opportunities, and mentorship programs. With access to these experiences becoming more constrained since the COVID-19 pandemic, our findings highlighted the perception among the premedical advisers who we interviewed that many PWI medical schools engaged little with HBCUs in close proximity, and even those that did engage at times may have done so superficially.

Not only may the proximity of PWIs in relation to large academic medical centers impact access to enrichment activities, but the long-standing history of institutional bias against HBCUs may also exacerbate the differential opportunities available to students. HBCUs, particularly public land-grant institutions designated under the Morrill Act of 1890, have faced funding disparities since their inception. According to the National Center for Education Statistics, multiple states underfunded 18 HBCUs by $12.8 billion from 1987 to 2020.^28,29^ These differences between PWIs and HBCUs suggest a manifestation of historic and ongoing institutional bias. Additionally, HBCUs often have smaller endowments than PWIs.^30^ These funding gaps have led to decreased funding for infrastructure, faculty salaries, and student grants, burdening the institutions with more debt and requiring students to take out more student loans to further their education.^29,31^

Our findings highlighted premedical advisers’ resourcefulness and creativity in finding the academic enrichment activities needed to help students become competitive medical school applicants despite these challenges. Alternative strategies, such as dual enrollment in interdisciplinary clinical opportunities and institutional-required internships through local partnerships, may offer opportunities for HBCU students to obtain valuable shadowing experiences. HBCUs may seek to establish partnerships with local medical specialty chapters and minority medical organizations (eg, the National Medical Association) to help systematize access opportunities for students.^32^

At an interpersonal level, existing literature highlights the importance of family support along the journey to becoming a physician. In a qualitative study of Black male medical students and physicians that explored contributors to their success in admission to and graduation from medical school, participants emphasized the role of family support throughout their education as particularly important.^33^ Black medical students also highlighted lack of support from family as a source of stress due to a lack of understanding of the medical school experience, low resources, and an inability to offer financial support.^34^ Our study provides insight into how HBCU premedical advisers reported leveraging family involvement and peer support as an advising strategy to help motivate and keep students accountable to their goals through role modeling and messages of encouragement.

### Limitations

Our findings should be considered in the context of several limitations. The premedical advisers in the sample may not represent the views and insights of all HBCU premedical advisers. Data on premedical student enrollment, medical school acceptance rates, and type of medical school attended for graduates (PWIs vs HBCUs) were not available to the study team. Because we focused on premedical advisers from HBCUs, our findings may not be generalizable to the experience of all Black premedical undergraduates. Our study also drew on experiences of premedical advisers during the COVID-19 pandemic, which may have adversely affected students’ successful navigation to medical school.^35^

## Conclusions

This qualitative study highlighted the opportunities and challenges that HBCU students faced when navigating the journey to medical school from the perspective of premedical advisers. HBCUs are important to the growth of Black physicians in the US by producing a vast number of medical school applicants and by training physicians. By not addressing barriers for undergraduate students at HBCUs, medical schools and the medical field at large may be missing critical opportunities to welcome unique insights into their communities and the field of medicine. Future studies could compare and contrast the perspectives of premedical advisers at PWIs in the context of Black undergraduate students navigating to medical school as well as the perspectives of Black students seeking admission to medical school from differing institutions (PWIs vs HBCUs).

## Notes

Supplement.
Data Sharing Statement

## References

[1] Jang CJ, Lee HC. A review of racial disparities in infant mortality in the US. Children (Basel). 2022;9(2):257. doi:10.3390/children9020257 35204976 PMC8870826

[2] Huang RS, Spence AR, Abenhaim HA. Racial disparities in national maternal mortality trends in the United States from 2000 to 2019: a population-based study on 80 million live births. Arch Gynecol Obstet. 2024;309(4):1315-1322. doi:10.1007/s00404-023-06999-6 36933039

[3] Greenwood BN, Hardeman RR, Huang L, Sojourner A. Physician-patient racial concordance and disparities in birthing mortality for newborns. Proc Natl Acad Sci U S A. 2020;117(35):21194-21200. doi:10.1073/pnas.1913405117 32817561 PMC7474610

[4] Snyder JE, Upton RD, Hassett TC, Lee H, Nouri Z, Dill M. Black representation in the primary care physician workforce and its association with population life expectancy and mortality rates in the US. JAMA Netw Open. 2023;6(4):e236687. doi:10.1001/jamanetworkopen.2023.6687 37058307 PMC10105312

[5] Xierali IM, Nivet MA. The racial and ethnic composition and distribution of primary care physicians. J Health Care Poor Underserved. 2018;29(1):556-570. doi:10.1353/hpu.2018.0036 29503317 PMC5871929

[6] Tipirneni R, Kieffer EC, Ayanian JZ, . Factors influencing primary care providers’ decisions to accept new Medicaid patients under Michigan’s Medicaid expansion. Am J Manag Care. 2019;25(3):120-127. 30875180 PMC7169442

[7] Kington R. Increasing racial and ethnic diversity among physicians: an intervention to address health disparities? In: Smedley BD, Stith AY, Colburn L, Evans CH; Institute of Medicine (US), eds. The Right Thing to Do, the Smart Thing to Do: Enhancing Diversity in the Health Professions: Summary of the Symposium on Diversity in Health Professions in Honor of Herbert W. Nickens. National Academies Press; 2001. 25057572

[8] Association of American Medical Colleges. Physician specialty data report: active physicians who identified as Black or African-American. 2021. Accessed August 30, 2023. https://www.aamc.org/data-reports/workforce/data/active-physicians-black-african-american-2021

[9] Association of American Medical Colleges. Underrepresented in medicine definition. Accessed July 30, 2024. https://www.aamc.org/what-we-do/equity-diversity-inclusion/underrepresented-in-medicine

[10] US Census Bureau. Quick facts: United States. Accessed August 30, 2023. https://www.census.gov/quickfacts/fact/table/US/PST045222

[11] Chandra R, Dunlap CE. SCOTUS deals a blow to American diversity by overturning affirmative action. Psychiatr News. 2023;58(8). doi:10.1176/appi.pn.2023.08.8.59

[12] Barzansky B, Catanese VM; Liaison Committee on Medical Education. Response letter to the Committee on Education and the Workforce, US House of Representatives. Accessed August 23, 2024. https://lcme.org/wp-content/uploads/2023/05/LCME-Response-to-Committee-on-Education-and-the-Workforce.pdf

[13] Ly DP, Essien UR, Olenski AR, Jena AB. Affirmative action bans and enrollment of students from underrepresented racial and ethnic groups in U.S. public medical schools. Ann Intern Med. 2022;175(6):873-878. doi:10.7326/M21-4312 35500257

[14] Gasman M, Smith T, Ye C, Nguyen TH. HBCUs and the production of doctors. AIMS Public Health. 2017;4(6):579-589. doi:10.3934/publichealth.2017.6.579 30155503 PMC6111265

[15] Shuler HD, Spencer EC, Davis JS, . Learning from HBCUs: how to produce Black professionals in STEMM. Cell. 2022;185(16):2841-2845. doi:10.1016/j.cell.2022.06.013 35716668 PMC9376858

[16] Rodríguez JE, López IA, Campbell KM, Dutton M. The role of historically Black college and university medical schools in academic medicine. J Health Care Poor Underserved. 2017;28(1):266-278. doi:10.1353/hpu.2017.0022 28239001

[17] United Negro College Fund. By the numbers: how HBCUs stack up. Accessed March 20, 2024. https://uncf.org/the-latest/by-the-numbers-how-hbcus-stack-up

[18] Association of American Medical Colleges. Diversity in medicine: facts and figures 2019. Table 10. U.S. medical school Black or African American graduates (alone or in combination) from historically Black colleges and universities (HBCUs), 1978-1979 through 2018-2019. Accessed September 7, 2023. https://www.aamc.org/data-reports/workforce/data/table-10-us-medical-school-black-or-african-american-graduates-alone-or-combination-historically

[19] Steele D. Will more medical schools mean more Black doctors? *Inside Higher Ed*. May 12, 2022. Accessed September 7, 2023. https://www.insidehighered.com/news/2022/05/13/new-med-schools-planned-need-black-doctors-continues

[20] Palinkas LA, Horwitz SM, Green CA, Wisdom JP, Duan N, Hoagwood K. Purposeful sampling for qualitative data collection and analysis in mixed method implementation research. Adm Policy Ment Health. 2015;42(5):533-544. doi:10.1007/s10488-013-0528-y 24193818 PMC4012002

[21] Tong A, Sainsbury P, Craig J. Consolidated criteria for reporting qualitative research (COREQ): a 32-item checklist for interviews and focus groups. Int J Qual Health Care. 2007;19(6):349-357. doi:10.1093/intqhc/mzm042 17872937

[22] Hennink MM, Kaiser BN, Marconi VC. Code saturation versus meaning saturation: how many interviews are enough? *Qual Health Res*. 2017;27(4):591-608. doi:10.1177/1049732316665344 PMC9359070 27670770

[23] Thomas DR. A general inductive approach for analyzing qualitative evaluation data. Am J Eval. 2006;27(2):237-246. doi:10.1177/1098214005283748

[24] Boatright DH, Samuels EA, Cramer L, . Association between the Liaison Committee on Medical Education’s diversity standards and changes in percentage of medical student sex, race, and ethnicity. JAMA. 2018;320(21):2267-2269. doi:10.1001/jama.2018.13705 30512090 PMC6583477

[25] Thornhill T. Racial salience and the consequences of making White people uncomfortable: intra-racial discrimination, racial screening, and the maintenance of White supremacy. Sociol Compass. 2015;9(8):694-703. doi:10.1111/soc4.12287

[26] Worrell FC, Mendoza-Denton R, Vandiver BJ, Fhagen PE, Cross WE Jr. Incorporating a race salience subscale into the cross racial identity scale (CRIS). J Black Psychol. 2020;46(8):638-658. doi:10.1177/0095798420967598

[27] Thornhill T. We want Black students, just not you: how White admissions counselors screen Black prospective students. Sociol Race Ethnic. 2019;5(4):456-470. doi:10.1177/2332649218792579

[28] Allen WR, Devost A, Mack C. Hidden in plain sight: historically Black colleges and universities in America. Quad Sociol. 2020;83(LXIV):25-46. doi:10.4000/qds.4044

[29] Adams S, Tucker H. How America cheated its Black colleges. *Forbes*. Updated September 22, 2022. Accessed August 23, 2024. https://www.forbes.com/sites/susanadams/2022/02/01/for-hbcus-cheated-out-of-billions-bomb-threats-are-latest-indignity/

[30] Hamilton D, Darity WA Jr. The political economy of education, financial literacy, and the racial wealth gap. Fed Reserve Bank St. Louis Rev. 2017;99(1):59-76.

[31] The Institute for College Access & Success. Fact sheet: race and economic mobility—college value at historically Black colleges and universities. February 7, 2024. Accessed August 11, 2024. https://ticas.org/racial-equity-agenda/race-and-economic-mobility-college-value-at-historically-black-colleges-and-universities-2/

[32] Mitchell EP. The National Medical Association 1895-2020: struggle for healthcare equity in the United States of America. J Natl Med Assoc. 2020;112(4):331-332. doi:10.1016/j.jnma.2020.08.001 32891361 PMC7467650

[33] Thomas B, Manusov EG, Wang A, Livingston H. Contributors of Black men’s success in admission to and graduation from medical school. Acad Med. 2011;86(7):892-900. doi:10.1097/ACM.0b013e31821d6f3d 21617511

[34] Odom KL, Roberts LM, Johnson RL, Cooper LA. Exploring obstacles to and opportunities for professional success among ethnic minority medical students. Acad Med. 2007;82(2):146-153. doi:10.1097/ACM.0b013e31802d8f2c 17264692

[35] Weiss J, Holaday L, Keene D, . Perspectives of historically Black college and university advisors to premedical students during the COVID-19 pandemic: a qualitative study. JAMA Netw Open. 2022;5(10):e2238563. doi:10.1001/jamanetworkopen.2022.38563 36269351 PMC9587479

