# Error in the Introduction

**DOI:** 10.1001/jamanetworkopen.2025.0790

**Published:** 2025-02-11

In the Original Investigation titled “Perspectives on Medical School Admission for Black Students Among Premedical Advisers at Historically Black Colleges and Universities,”^1^ published October 23, 2024, in the fourth paragraph of the Introduction, the first sentence was adjusted to state “HBCUs” instead of “HBCU medical schools” and the second sentence was adjusted to specify that 9.8% was the percentage of Black medical school graduates in 2019 who completed their undergraduate training at HBCUs, not training at HBCU medical schools. Also, citations 18 and 19 were added to the first sentence of this paragraph, with only reference 18 cited for the second sentence. This article has been corrected.^1^

## References

[1] Weiss J, Nguementi Tiako MJ, Akingbesote ND, . Perspectives on medical school admission for Black students among premedical advisers at Historically Black Colleges and Universities. JAMA Netw Open. 2024;7(10):e2440887. doi:10.1001/jamanetworkopen.2024.40887 39441593 PMC11581641

