# Error in the Byline

**DOI:** 10.1001/jamanetworkopen.2026.15834

**Published:** 2026-05-08

In the Original Investigation titled “Perspectives on Medical School Admission for Black Students Among Premedical Advisers at Historically Black Colleges and Universities,” published on October 23, 2024, the surname of second author Max Jordan Nguemeni Tiako, MD, MS, was misspelled. This article has been corrected online.^1^
