# Supplementary material for: Perspectives on Medical School Admission for Black Students Among Premedical Advisers at Historically Black Colleges and Universities
Source: JAMA Netw Open. 2024 Oct 23;7(10):e2440887. doi: 10.1001/jamanetworkopen.2024.40887 (PMC11581641; doi:10.1001/jamanetworkopen.2024.40887)
Supplement: Supplement. — Data Sharing Statement [file jamanetwopen-e2440887-s001.pdf]

Data Sharing Statement

Weiss. Perspectives on Medical School Admission for Black Students Among Premedical Advisers at HBCUs. *JAMA Netw Open*. Published October 23, 2024.
doi:10.1001/jamanetworkopen.2024.40887

Data
Data available: No

Additional Information
Explanation for why data not available: qualitative study
